# Dupilumab and ritlecitinib combination therapy in a patient with alopecia totalis and atopic disease

**DOI:** 10.1016/j.jdcr.2026.06.044

**Published:** 2026-06-25

**Authors:** Isabella Zappi, Archie Spindler, Derek Maas, Seth J. Orlow, Jerry Shapiro, Kristen I. Lo Sicco

**Affiliations:** a The Ronald O. Perelman Department of Dermatology, NYU Grossman School of Medicine, New York, New York; b University of Rochester School of Medicine, Rochester, New York

**Keywords:** alopecia areata, alopecia totalis, atopic dermatitis, baricitinib, combination therapy, deuruxolitinib, dupilumab, Janus kinase inhibitor, ritlecitinib

## Introduction

The management of alopecia areata (AA) has revolutionized in the past decade with the advent of Janus kinase inhibitors (JAKi), offering a novel therapeutic solution. Now 3 oral JAKi—baricitinib, ritlecitinib, and deuruxolitinib—have been Food and Drug Administration approved in the United States for the treatment of AA with evidence in the literature demonstrating efficacy of additional JAKi including oral tofacitinib and upadacitinib.^1^

Oral JAKi have demonstrated superior efficacy in AA compared to placebo in multiple trials, with baricitinib, ritlecitinib, and deuruxolitinib achieving Severity of Alopecia Tool (SALT) scores of ≤20 in 23% to 38.8% of patients at 24-36 weeks of treatment.^2^, ^3^, ^4^ Dupilumab, while Food and Drug Administration approved for various type 2 immune-mediated diseases, is not approved for AA.^1^ However, a Phase 2a randomized controlled trial exhibited AA stabilization at 24 weeks and continued SALT score improvement thereafter, particularly among patients with baseline immunoglobulin E levels ≥200 IU/mL, which predicted treatment response with 83% accuracy.^5^

Limited data exist on JAKi-dupilumab combination therapy for the treatment of AA. Here, we present a case of a child with alopecia totalis (AT) and concomitant AD who had a partial response to monotherapy and subsequent improved response to combination therapy.

## Case report

**Fig 1:**
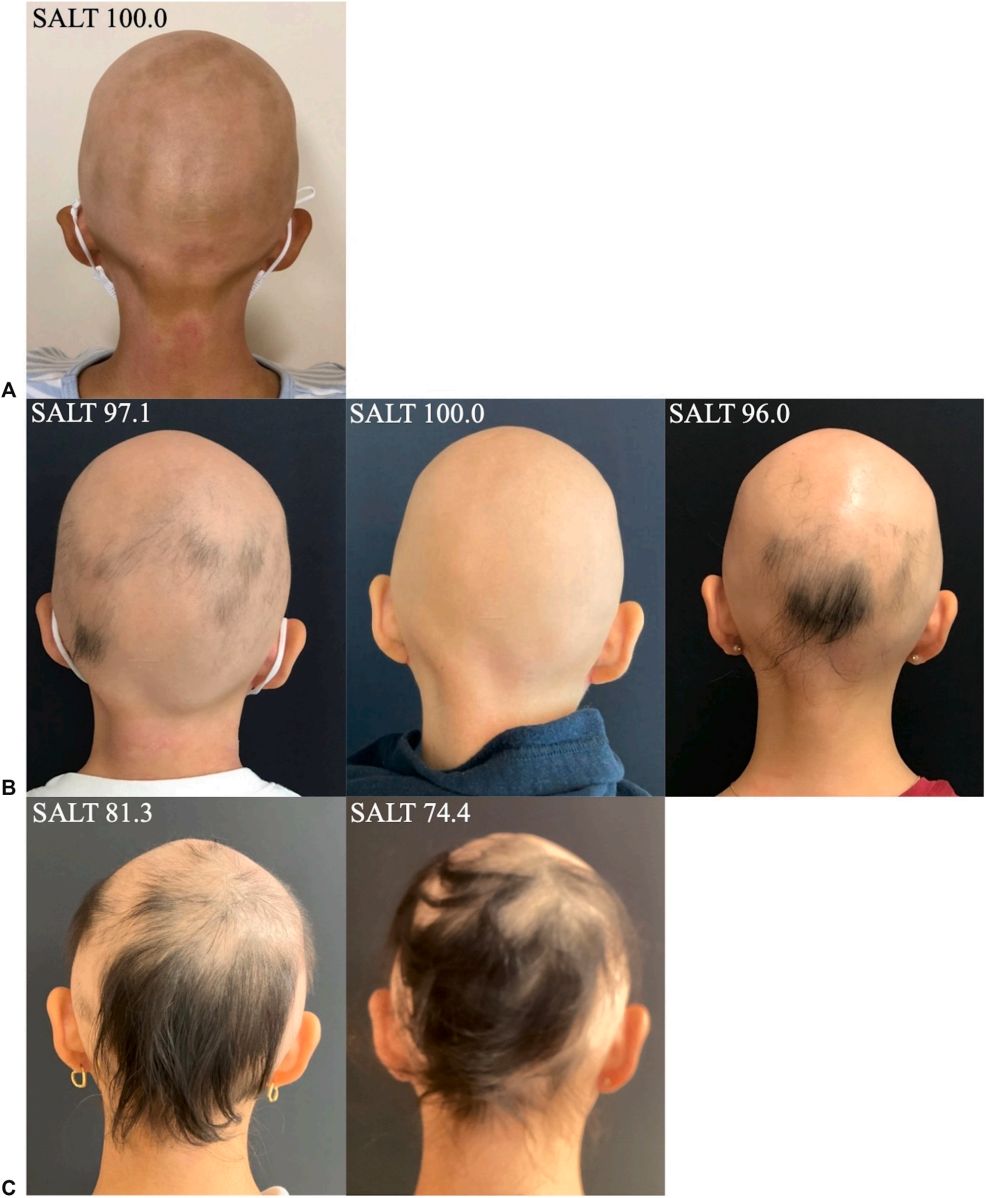
Alopecia totalis baseline **(A)** dupilumab monotherapy **(B)** and ritlecitinib monotherapy **(C)** scalp photographs with corresponding Severity of Alopecia Tool scores in a female pediatric patient. Baseline SALT score was 100.0 **(A)**; dupilumab monotherapy SALT scores were 97.1, 100.0, and 96.0 at 6 months, 12 months, and >2 years **(B)**; and ritlecitinib monotherapy SALT scores were 81.3 and 74.4 at 6 months and 12 months **(C)**. *SALT*, Severity of Alopecia Tool.

We present the case of a 15-year-old female diagnosed with AT at age 5 with concomitant mild AD and allergic rhinitis. Prior unsuccessful therapies for AT included diphencyprone contact sensitization therapy, topical tofacitinib, oral prednisone, topical ruxolitinib, narrow-band ultraviolet-B excimer laser (308 nm), and topical fluocinonide and pimecrolimus. With a baseline SALT score of 100.0 at 10 years of age, she was initiated on dupilumab (600 mg loading dose, followed by 300 mg every 4 weeks, then appropriately weight adjusted to 200 mg every 2 weeks) (Fig 1, *A*; Table I). Dupilumab monotherapy resulted in minimal regrowth, with SALT scores of 97.1, 100.0, and 96.0 at 6 months, 12 months, and >2 years, respectively, while her AD cleared (Fig 1, *B*). Dupilumab was discontinued and ritlecitinib 50 mg daily was initiated for her AT which achieved partial improvement with SALT scores of 81.3 and 74.4 after 3 and 12 months of therapy, respectively; however, her AD began to flare (Fig 1, *C*).

**Table I:** Treatment drug, dose, and frequency as well as pretreatment, monotherapy, and combination therapy Severity of Alopecia Tool (SALT) scores

Therapy	Dose and frequency	Treatment Time	SALT
Pretreatment	—	—	100.0
Dupilumab	200 mg every 2 wk	6 mo	97.1
		12 mo	100.0
		>2 yr	96.0
Ritlecitinib	50 mg daily	3 mo	81.3
		12 mo	74.4
Ritlecitinib + Dupilumab	50 mg daily; 200 mg every 2 wk	6 mo	53.1
		12 mo	19.6

**Fig 2:**
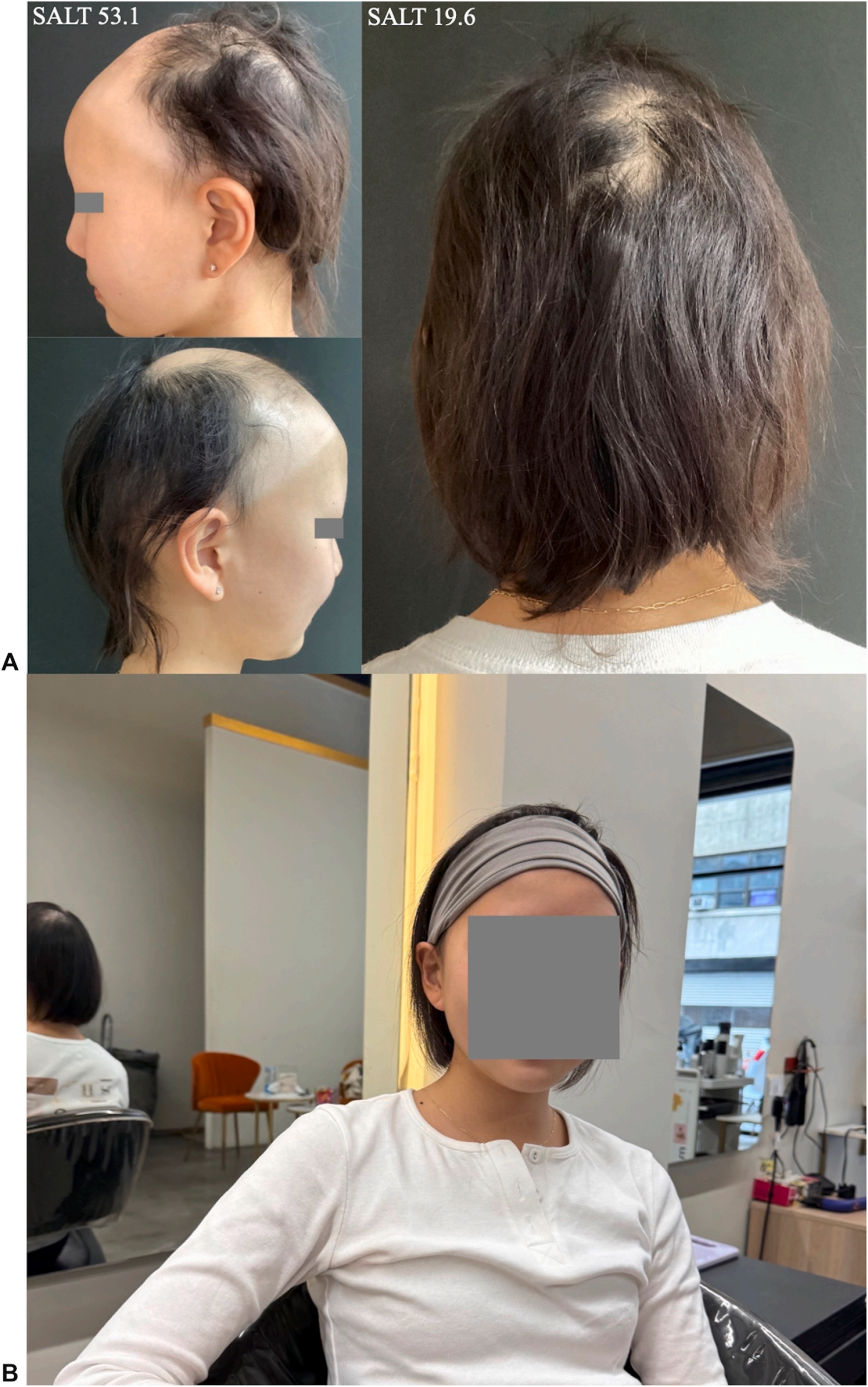
Alopecia totalis dupilumab and ritlecitinib combination therapy scalp photographs with corresponding Severity of Alopecia Tool (SALT) scores in a female pediatric patient. Combination therapy SALT scores were 53.1 and 19.6 at 6 and 12 months **(A)**. Patient receiving her first haircut in over a decade after 12 months of combination therapy **(B)**. *SALT*, Severity of Alopecia Tool.

Given persistent AA and worsening AD, dupilumab was reinitiated after 12 months of ritlecitinib monotherapy to optimize AA and AD control. While JAKi targeting both AD and AA—particularly JAK1 inhibitors such as upadacitinib or baricitinib—were considered, reinitiation of dupilumab was favored based on patient and family preference, prior tolerability to both ritlecitinib and dupilumab, favorable safety profiles, and the goal of maximizing hair regrowth, which we were concerned might not be adequately achieved with JAK pathway inhibition alone. Dupilumab and ritlecitinib combination therapy led to marked hair regrowth with a SALT score improvement to 53.1 and 19.6 after 6 and 12 months, respectively, and AD improvement (Fig 2, *A*). Comparatively, maximum SALT score reductions after 12 months were 2.9 with dupilumab monotherapy, 21.6 with ritlecitinib monotherapy, and 54.8 with dupilumab and ritlecitinib combination therapy (Table II). No adverse events were reported throughout treatment. While each monotherapy produced a partial response, combination therapy provided additional benefit. Notably, the patient received her first haircut in over a decade while on combination therapy (Fig 2, *B*).

**Table II:** Monotherapy and combination therapy Severity of Alopecia Tool (SALT) maximum score reductions after 12 months of therapy

	SALT score
Dupilumab monotherapy maximum SALT score reduction	2.9
Ritlecitinib monotherapy maximum SALT score reduction	21.6
Dupilumab and ritlecitinib combination therapy maximum SALT score reduction	54.8

*SALT*, Severity of alopecia tool.

## Discussion

There are currently limited data describing the use of JAKi-dupilumab combination therapy for AA. Prior reports describe patients with concomitant AA and AD who achieved partial improvement with JAKi or dupilumab monotherapy and subsequently switched therapies to optimize disease control. Seo et al described an adult with AT and severe AD who exhibited near-complete AD improvement but persistent AA on dupilumab who desired greater AA control and, after transitioning to baricitinib, obtained marked AA improvement.^6^ Liu et al presented a pediatric patient with severe AD refractory to dupilumab and mild AA who instead desired greater AD control and achieved significant improvement in AD and complete hair regrowth after switching to abrocitinib.^7^ Conversely, Cai et al described a pediatric patient with alopecia universalis refractory to baricitinib and concomitant allergic rhinitis who exhibited significant hair regrowth after switching to dupilumab.^8^ Notably, no significant adverse events were reported in these cases.^6^, ^7^, ^8^ Collectively, these cases elucidate the potentially complementary therapeutic roles of dupilumab and JAKi in patients with concomitant AA and AD, while highlighting the clinical challenge of balancing AD and AA disease control and optimizing treatment selection in this population.

Here, we present a case of a pediatric patient with AT and concomitant AD who experienced partial hair regrowth with dupilumab and ritlecitinib monotherapy and subsequently demonstrated additional improvement with combination therapy. This observation suggests that targeting distinct immunologic pathways may confer synergistic therapeutic benefits in AA, particularly in patients with disease refractory to monotherapy.

Distinct mechanisms of action are seen with JAKi and dupilumab when used in AA. JAKi block the JAK-signal transducer and activator of transcription pathway through inhibition of JAK1, JAK2, and/or JAK3 receptors.^1^ Cytokines implicated in AA pathogenesis—including interferon-γ, interleukin (IL)-2, IL-7, and IL-15—activate JAK receptors.^1^ Therefore, inhibition of this signaling cascade (through JAK3 in the case of ritlecitinib), which otherwise would lead to further gene expression with potential hair follicle destruction, is beneficial in AA.^1^ Meanwhile, dupilumab downregulates Th2-mediated inflammation through IL-4 and IL-13 signaling inhibition.^4^ While inflammation in AA has traditionally been viewed as a predominantly T helper 1-driven disease, emerging evidence has suggested that the Th2 pathway may additionally contribute through several mechanisms.^1^ Not only were Th2 cells found to be increased in the circulation of patients with AA, the Th2-derived IL-13 cytokine was also found to be a locus of genetic susceptibility for AA in several studies.^9^^,^^10^ Through mechanisms that likely involve modulation of the Th2 inflammatory pathway when the IL-4 receptor α subunit is blocked by dupilumab, the drug has shown modest efficacy when used for AA, especially in patients with concomitant AD or elevated immunoglobulin E levels.^4^

These findings suggest that dual targeting of JAK-signal transducer and activator of transcription and T helper 2 mediated inflammatory pathways with JAKi and dupilumab in AA may represent a promising bespoke therapeutic strategy for patients with refractory AA and coexisting atopic disease. Larger prospective cohort studies are warranted to further characterize outcomes and safety.

### Declaration of generative AI and AI-assisted technologies in the writing process

Not used.

## Conflicts of interest

Dr Orlow served as an advisory board member for Veradermics. Dr Shapiro is an investigator for Pfizer and a consultant for Pfizer, Lilly, Replicel Life Science, Regen Lab, Thirty Madison, DS Laboratories, Aclaris Therapeutics, Incyte, and Almirall. Dr Lo Sicco is a prior investigator for Pfizer and Regen Lab; consultant for Pfizer, Lilly, Priovant, Veradermics, Ro and Aquis; and board member for the Scarring Alopecia Foundation and American Hair Research Society. Authors Zappi, Spindler, and Maas have no conflicts of interest to declare.
